# Successful endourological treatment for a rare case of atazanavir‐induced urinary stones

**DOI:** 10.1002/iju5.12779

**Published:** 2024-08-30

**Authors:** Takehiro Nakamura, Ryoji Takazawa, Yosuke Umino

**Affiliations:** ^1^ Tokyo Metropolitan Otsuka Hospital Urology and Kidney Stone Center Toshima Tokyo Japan

**Keywords:** atazanavir, lithotripsy, ureteral calculi, ureteroscopy, urolithiasis

## Abstract

**Introduction:**

Atazanavir, an anti‐HIV drug, induces urinary stone formation. We herein report a case of atazanavir‐induced bilateral ureteral stones treated with endourological procedures.

**Case presentation:**

A 47‐year‐old male patient on atazanavir was admitted with right flank pain. The patient's serum creatinine level was 3.60 mg/dL. Plain computed tomography showed bilateral hydronephrosis. The left upper ureter was obstructed by a 13 × 12 × 9‐mm mass, which had very low CT attenuation values. The lower part of the right ureter was obstructed, but no mass was observed. Given his medical history, we suspected bilateral ureteral obstruction caused by drug‐induced stones. A ureteral stent was successfully inserted on the right side, but not on the left side. Thereafter, a bilateral ureteroscopic lithotripsy was performed. Infrared spectrophotometry revealed that the stone component was atazanavir.

**Conclusion:**

Understanding the characteristics of these rare drug‐induced stones will aid in the proper early diagnosis and treatment.

**Abbreviations & Acronyms**
AIDS: acquired immunodeficiency syndrome
CT: computed tomography

**Keynote message**

Atazanavir, a protease inhibitor used for HIV treatment, is known to induce urinary stones, which have atypical CT findings for urinary stones. Although rare, drug‐induced stones should be suspected in patients with a history of HIV treatment who present with hydronephrosis or renal colic without stones on imaging. Understanding the characteristics of these rare drug‐induced stones will aid in early diagnosis and treatment.

## Introduction

Drug‐induced stones can be classified into two types: those in which the drug itself directly forms crystals and stones and those in which the stones are formed due to metabolic changes caused by drug administration. The former case is extremely rare. We herein report a case of acute kidney injury caused by atazanavir‐induced bilateral ureteral stones that were treated with endourological procedures.

## Case presentation

**Fig. 1:**
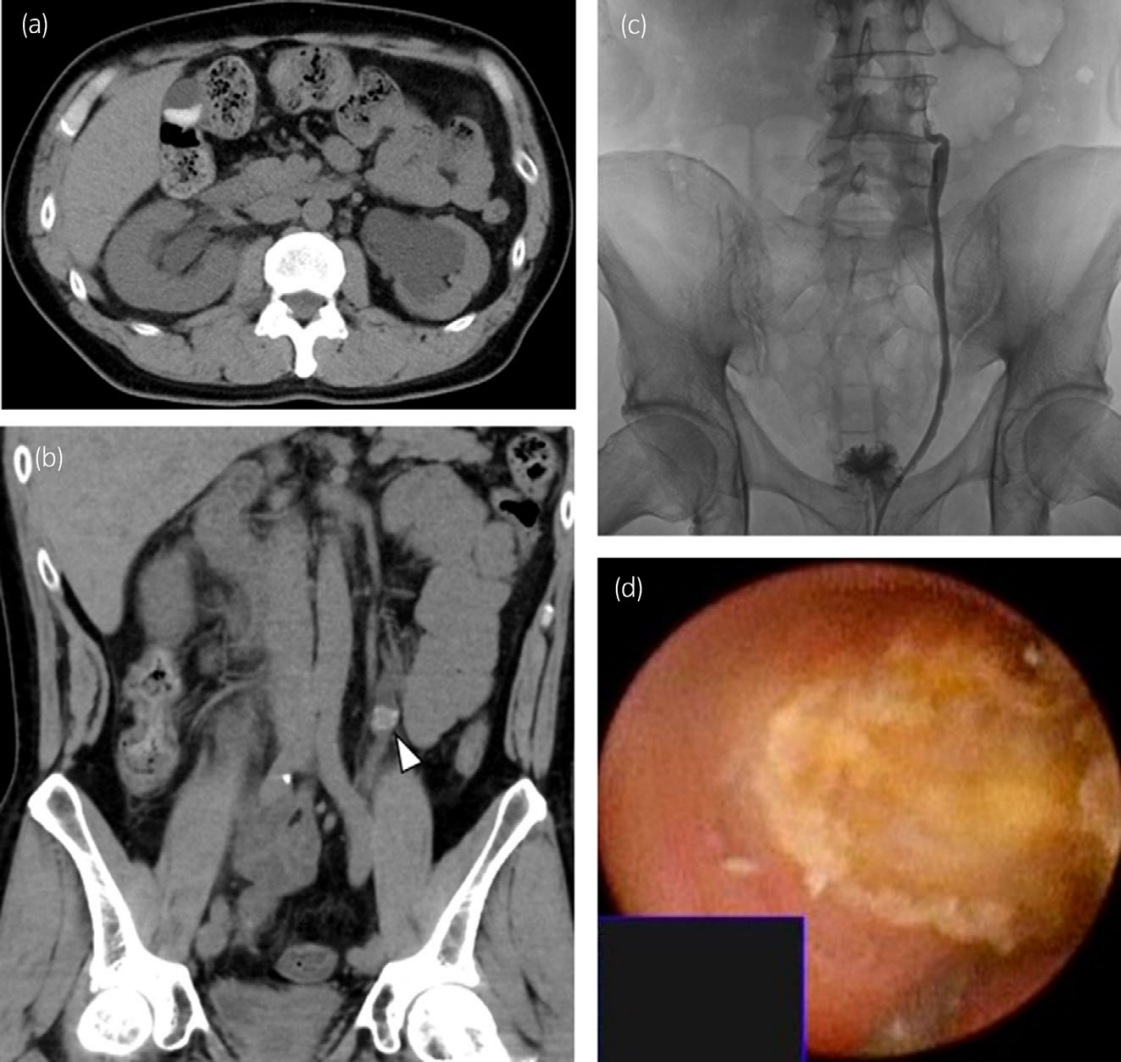
Plain CT images of the bilateral hydronephrosis with left‐sided predominance and thinning of the cortex of the left kidney (a) and the mass (arrowhead) in the left upper ureter (b). X‐ray image during retrograde ureterography. The contrast medium was stopped at the site of the mass (c). Endoscopic image of the left ureteral stone during lithotripsy (d).

**Fig. 2:**
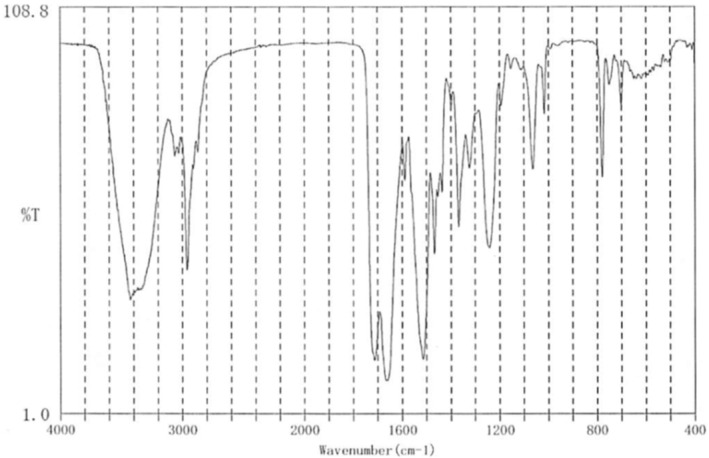
The wavelength pattern of the infrared spectrophotometry of the stone fragment which is consistent with that of drug atazanavir.

A 47‐year‐old male patient was referred to our hospital with a chief complaint of right flank pain. He was diagnosed with AIDS at 28 years of age and was receiving atazanavir (300 mg), ritonavir (100 mg), and abacavir/lamivudine (600/300 mg) combination tablets daily for over 10 years. He noticed decreased urinary output that persisted for 2 weeks. He had developed right back pain 1 week earlier, which later progressed to right flank pain, vomiting, and loss of appetite. He was found to have decreased renal function (serum creatinine level: 3.60 mg/dL). Urinalysis revealed a pH of 5.5, and examination of the urinary sediment showed 5–9 RBC and 30–49 WBC per high‐power field. The peripheral blood count and blood chemistry results were unremarkable, except for a high CRP level (9.43 mg/dL). Plain CT showed bilateral hydronephrosis with left‐sided predominance and thinning of the cortex of the left kidney (Fig. 1a). The right ureter showed no clear findings typical of stones, although the lower ureter showed obstruction. The left ureter showed an obstruction in the upper ureter with a mass measuring 13 × 12 × 9 mm, which was not a typical stone finding (Fig. 1b). The mean CT attenuation value in the region of interest of the mass was 59 (range: 16–96) HU. Given his history of taking the anti‐HIV drug atazanavir, we strongly suspected acute kidney injury due to ureteral obstruction from drug‐induced stones and performed an emergency ureteral stent insertion. The procedure was performed under fluoroscopic guidance, and a ureteral stent was inserted without any complications on the right side. However, on the left side, the guidewire could not pass through the site at which the obstruction was thought to have originated (no stone shadow could be seen), and we abandoned ureteral stent insertion (Fig. 1c). After the patient's renal function recovered to a serum creatinine level of 1.7 mg/dL, bilateral ureteroscopic lithotripsy was performed. We used a holmium YAG laser (Litho EVO®, Quanta System, Milan, Italy) to fragment the stones and a nitinol basket catheter (Escape®, Boston Scientific, MA, US) to remove stone fragments. After removal of the right ureteral stent, the right upper urinary tract was checked using semirigid and flexible ureteroscopy. Only a 2‐mm stone was found in the upper ureter, which crumbled when grasped using a basket catheter. Thereafter, a semirigid ureteroscope was inserted into the left ureter, and the impacted stone was reached. The stone was impacted in the ureteral wall with an edematous mucosa. The stone was yellowish in color, like a calcium dihydrate stone, but very fragile like struvite (Fig. 1d). Tiny fragments were selected for the stone component analysis. The surgery was completed by inserting ureteral stents bilaterally. The operative time was 107 min. Postoperatively, the renal function recovered to a serum creatinine level of 1.3 mg/dL. The wavelength pattern of infrared spectrophotometry of the stone fragment was consistent with that of the drug atazanavir (Fig. 2). Two weeks after surgery, bilateral ureteral stents were removed. Three months after surgery, plain CT showed no signs of new stones or hydronephrosis.

## Discussion

Drug‐induced stones are rare, accounting for 1%–2% of all urinary tract stones; however, in most cases, the condition is preventable and should be thoroughly investigated.^1^, ^2^

Anti‐HIV therapy is recommended for all patients with HIV infections. As the duration of treatment to eliminate latent HIV‐infected cells is very long, it is necessary to continue treatment for almost a lifetime to control the disease. Atazanavir and ritonavir are protease inhibitors, and the combination of these two drugs (ATV/r) is one of the most important first‐line drugs owing to its high efficacy and tolerability. However, owing to the risk of urinary tract stones and jaundice,^3^ it is not the current first‐line therapy. Nevertheless, it is still a drug that can be used as an alternative in situations in which current first‐line drugs are unavailable.

Hamada *et al*. investigated the incidence of ATV/r urolithiasis.^4^ They found an estimated incidence of 23.7 cases per 1000 person‐years among patients treated with ATV/r between January 2004 and June 2010, which was approximately 10 times higher than the estimated incidence of 2.20 cases per 1000 person‐years for other drugs. A significant relationship between ATV/r use and the occurrence of kidney stones was found, with a median time from the start of treatment to the diagnosis of kidney stones of 24.5 months.

However, the mechanism underlying atazanavir stone formation remains unclear. Atazanavir is primarily metabolized and excreted by the liver. However, after a single 400 mg dose in healthy subjects, up to 7% of the drug is excreted in urine.^3^ Stones may be related to the urinary precipitation of atazanavir.^5^, ^6^, ^7^ Most stones due to ATV/r are atazanavir stones, and stones primarily caused by ritonavir have been reported but are very rare.^8^ Atazanavir stones can be radiolucent on radiography or CT, making diagnosis difficult. Drug‐induced stones should be suspected when a patient shows hydronephrosis or renal colic without stones on imaging studies.^9^, ^10^

In the present case, there were no obvious stones in the bilateral ureters at first glance. Upon closer examination, a very low‐density shadow with low CT value was observed in the left ureter. Since this patient had been taking the drug for approximately 10 years, we do not know when urolithiasis began to form. Given the progressive hydronephrosis on the left side, we expect that the left ureteral stone had been present there for a long time. An accidental attack of ureteral stones on the right side ultimately led to a diagnosis. Although the number of patients using ATV/r is expected to decrease in the future, drug‐induced stones should be suspected in patients with a history of HIV treatment, who present with hydronephrosis or renal colic without typical stone findings on imaging. Understanding the characteristics of these rare drug‐induced stones will aid in the early diagnosis and treatment.

## Author contributions

Takehiro Nakamura: Visualization; writing – original draft; writing – review and editing. Ryoji Takazawa: Supervision; visualization; writing – original draft; writing – review and editing. Yosuke Umino: Writing – review and editing.

## Conflict of interest

The authors declare no conflicts of interest in association with the present study.

## Approval of the research protocol by an Institutional Reviewer Board

Not applicable.

## Informed consent

Informed consent was obtained from the patient.

## Registry and the Registration No. of the study/trial

Not applicable.
